# Impact of the COVID-19 pandemic on clinical care and patient-focused outcomes of advanced nursing practice: A cross-sectional study

**DOI:** 10.1371/journal.pone.0313751

**Published:** 2024-11-14

**Authors:** Sek Ying Chair, Kelley Kilpatrick, Catrina Heffernan, Stacia M. Hays, Huaping Liu

**Affiliations:** 1 Nethersole School of Nursing, Faculty of Medicine, The Chinese University of Hong Kong, Hong Kong SAR, Hong Kong; 2 Ingram School of Nursing, McGill University, Montreal, QC, Canada; 3 Department of Nursing and Healthcare Sciences, Munster Technological University, Tralee, Ireland; 4 Louise Herrington School of Nursing, Baylor University, Dallas, Texas, United States of America; 5 School of Nursing, Peking Union Medical College, Beijing, China; Sungkyunkwan University - Suwon Campus: Sungkyunkwan University - Natural Sciences Campus, REPUBLIC OF KOREA

## Abstract

**Background:**

The COVID-19 pandemic has significantly impacted advanced practice nurses’ practice and posed great challenges in patient care delivery.

**Purpose:**

The aim of this study was to investigate the impact of the COVID-19 pandemic on the practice of advanced practice nurses in mainland China and Hong Kong Special Administrative Region (SAR).

Methods

A cross-sectional descriptive survey was conducted March 2021 and January 2022. Advanced practice nurses were invited to participate in an online survey. The questionnaire described the socio-demographic characteristics, the impact of the COVID-19 pandemic on advanced nursing practice, patient outcomes, education needs about COVID-19, and the challenges, support, and concerns related to the advanced practice nurse practice during the pandemic. Wilcoxon signed-rank test or McNemar test were applied to measure the practice of APNs before and during the COVID-19 pandemic.

**Results:**

Respondents **(**N **=** 336) were from mainland China (*n* = 234) and Hong Kong SAR (*n* = 102). Participants reported increased practice-related workload during the pandemic. The proportions of advanced practice nurses focused on disease prevention (36.9%) and psychosocial well-being (15.5%) for patient-focused outcomes during the pandemic were higher compared to before the pandemic. Key challenges and concerns during the pandemic included heavy workloads and health concerns for themselves and their families. Despite difficulties, there were reports of positive changes since the outbreak including implementation of innovative measures to facilitate the advanced practice nursing and education about COVID-19.

**Conclusion:**

The study findings highlight that advanced practice nurses’ work and responsibilities have changed in response to the pandemic. Providing education about COVID-19, innovative measures to facilitate advanced practice nursing, and understanding advanced practice nurses’ concerns and challenges in providing patient care may inform future developments for improving their professional practice.

## Introduction

The emergence of coronavirus disease (COVID-19) caused by the SARS-CoV-2 virus in late 2019, along with its rapid spread worldwide has led to millions of lives lost and severe strain on health systems [1, 2]. There have been over 772 million confirmed cases of COVID-19 across the globe with approximately seven million deaths as of December 17, 2023 [3]. Healthcare professionals play a vital role in the preparedness and response efforts for global health crisis, as demonstrated during the COVID-19 pandemic. As of May 2022, more than 180,000 healthcare professionals died from the COVID-19 [4]. Healthcare professionals were in close contact with patients with COVID-19, causing them to have a higher risk of being infected. As such, nurses were particularly vulnerable due to the close proximity and direct contact when caring for patients with COVID-19 [5]. To respond to the pandemic, nurses work across hospitals, communities, nursing homes, and governmental healthcare organizations and have a critical function in the healthcare system [6].

There was a global concern about the experience and impact of COVID-19 on nurses who are the largest group of frontliners of health systems [7, 8]. There were emergency regulatory changes and policy expansion to address the healthcare workforce needs during the COVID-19 pandemic leading to expansion of the scope of practice for advanced practice nurses (APNs) and creating unprecedented opportunities for APNs, particularly in the United States [9]. Many APNs had to change from their APN specialty to providing care for patients diagnosed with COVID-19 [10, 11]. With the shortage of providers and the need for increased access to healthcare, APNs are vital to maintaining functioning healthcare systems worldwide [12]. However, internationally, challenges associated with the changes in practice related to COVID-19 affected APNs ability to provide effective care. A cross-sectional study in the United Kingdom (UK) investigated the experiences of 124 APNs during the COVID-19 pandemic. Results showed that 43% of respondents considered leaving their job in the previous three months. APNs expressed concerns about inadequate protective equipment, protecting their health and that of their family members [11]. Another survey involving over 7,000 APNs in the United States (US) found that the pandemic affected many aspects of APNs’ practice, including scheduling changes, fewer new patient visits, fewer scheduled acute care visits, and less revenue for practices or facilities [10].

In the past, the first SARS outbreak in Asia in 2003 spread rapidly and intensely, leaving the health systems unprepared [13, 14]. During this outbreak, eight healthcare workers in Hong Kong died and there was evidence of distress (e.g., anxiety, fear, and depression) among front-line healthcare workers in this region [15]. Cooper et al. [16] found that 70% of the healthcare staff infected were nurses. This sample included three hospitals in mainland China. Nurses from mainland China expressed personal physical (e.g., protective equipment issues) and emotional challenges (patient demise, uncertainty about the contagion) while caring for patients with SARS [17]. Chen et al. [18] proposed that SARS prevention programmes that include in-service trainings, comprehensive human resource allocation, sufficient personal protective equipment, and accessibility of a mental health team might help the nurses to cope better when facing an unknown infectious disease in the future. Similarly, the above-mentioned impact of COVID-19 on APNs in the Western and Asian counterparts was alarming. Importantly, there was a lack of information related to these issues among APNs in the Asia region during the COVID-19 pandemic, especially from the countries or regions that had previous experience in dealing with the severe acute respiratory symptoms coronavirus (SARS) in 2003 such as mainland China and Hong Kong as a special administrative region of China but with higher influence from western culture. It is important to study the changes in roles and responsibilities among APNs particularly in the Asia region as the development of APNs in Asia has been relatively slower compared to their counterparts in Western countries.

The development and advancement of APN roles are in early stages in mainland China, with a lack of defined description for their role [19]. Despite the need to develop APNs in mainland China where a pioneer project (a 22-month APN postgraduate program) was conducted in Guangzhou, China between 2004 and 2005, the development of advanced practice nursing encountered numerous pushing factors including unclear role definition, lack of recognition from the public and other healthcare professionals, and lack of legal protection for APNs [20]. Nonetheless, the Development Plan for Nursing in China (2016–2020) stressed the need to comprehensively develop specialty nursing [21]. Despite lack of a national standard for the criteria of an APN in mainland China, the required qualifications include nurses who have obtained a master degree in nursing and an advanced certificate in a specialised area, and years of working experiences in specialty areas [22].

Meanwhile in Hong Kong SAR, the development of advanced nursing practice has been progressive. The first group of clinical nurse specialists was introduced in 1993 in Hospital Authority (HA) and this title was subsumed under the generic work title of APN in 2003. In 2008, the position of nurse consultant was introduced as a level above APN [21]. Nurse consultants play important role in healthcare by providing complex patient care and having diverse range of roles and responsibilities including managing complex cases, developing and planning of health services, ensuring quality of care and patient safety, educating and mentoring healthcare professionals, and involving in research activities [23]. There is a lack of clarity in the definition of roles and scope of practice among APNs [24], making it essential to have a study particularly in countries or regions in the early stage of APN role development. The findings can be used to build preliminary databases for describing APN roles and scope of practice, where their practice can be acknowledged and supported.

In addition, high degree of uncertainties of the COVID-19 on its characteristics, transmission dynamics, and effective measures to treat resulting infections made it necessary to study the impact of this pandemic on APNs who have expanded clinical capabilities. With the overwhelming COVID-19 demands, job demands, and resources on strain as above-mentioned during the unprecedented nature of the novel coronavirus, it was important to learn from what APNs had experienced particularly related to clinical care and patient-focused outcomes, and understanding the changes in APNs’ practice and roles from multiple perspectives before and after the COVID-19 pandemic. There is also a need to investigate the challenges and concerns APNs faced, and the activities they undertook during the pandemic in order to improve APNs’ practice. This study could help to provide insights into improvement opportunities, allowing healthcare professionals including APNs to be more prepared and resilient in facing future public health threats. In addition, findings from this study could further shape APN practice and develop strategies to effectively support APNs responding to future crisis situations.

## Methods

### Study design and participants

This was a cross-sectional descriptive study using quantitative method. Advanced practice nurses working in Hong Kong SAR and mainland China were recruited in an online survey using snowball sampling between March 2021 and January 2022. The criterion included was a nurse who was practicing in an APN role during the COVID-19 pandemic. In this study, an APN was defined as any nurse who was working in an advanced nursing practice role, clinical or non-clinical, requiring expert nursing knowledge and skill [25].

### Measures

The questionnaire was developed from the literature by a panel of eight nursing experts comprising of academics and practitioners in the field. The questionnaire included four sections: (1) sociodemographic data (nine items), (2) the impact of COVID-19 pandemic on the APN practice [five items]), (3) the impact of pandemic on patient and nurse outcomes (three items) and education about the COVID-19 pandemic (two items), and (4) challenges, support, and concerns in APN practice during the COVID-19 pandemic (five items). The impact of COVID-19 consists of items such as, ‘Which of the following was your major scope of practice before the COVID-19 pandemic?’ and ‘Which of the following is your major scope of practice during the COVID-19 pandemic?’ Participants were given two options including ‘direct care’ or ‘indirect care.’ Under each option, participants had to further answer the following: ‘if so, how many hours did/do you spend on clinical practice (hours/week)?’ ‘What is the nurse-patient ratio in your practice setting?’ and ‘How many patients did/do you need to take care of per shift?’ One of the items of the impact of pandemic on patient and nurse outcomes is ‘What were/are your focused patient outcomes before/during the COVID-19 pandemic?’ Participants were given six options including: ‘disease prevention’, ‘symptom management’, ‘psychosocial well-being’, ‘patient/family satisfactory’, ‘cost of care’, and ‘others’.

The contents of the questionnaire were critically commented and endorsed by the International Council of Nurses Practitioner/Advanced Practice Nurse (ICN NP/APNN) expert panel, which ensured the questionnaire covered the full range of dimensions related to APN practice (e.g., the responsibilities and activities such as direct and indirect clinical care). Subsequently, a panel of experts which included six advanced practice nurses conducted a content validity assessment on the questionnaire. Content validity provides evidence on the extent to which the items of the assessment instrument adequately represent the construct for a specific assessment purpose [26]. In this study, a total of 15 items (excluding the sociodemographic data) were included for the content validity assessment. The assessment was based on three areas: relevancy, clarity, and simplicity. According to Lynn [27], the cut-off point for item level CVI (I-CVI) is 0.78. In this questionnaire, the scores for I-CVI for all items fulfilled the criterion as they ranged between 0.83 and 1.0. In addition, the overall instrument-CVI, Average-CVI (Ave-CVI) is calculated as the average proportion of items rated as 3 or 4 among a panel of experts [28], where the acceptable standard of Ave-CVI ranges from 0.8 to 0.9 [26]. The Ave-CVI for this questionnaire ranged between 0.95 to 1.0 for relevancy, clarity, and simplicity.

### Sample size

As the participants of the web-based survey were recruited primarily via snowball sampling. We anticipated to recruit at least 100 participants from each study group. Such a sample size could enable to detect within-subject changes on the outcome responses with a small to moderate effect size of dz = 0.3 [29] with 80% power at a 5% level of significance (2-sided) by using Wilcoxon signed-rank test. The power analysis was conducted using GPower 3.1 [30]. A total of 336 APNs participated in this study.

### Data collection

The questionnaire was disseminated online. The aims and objectives of the study were clearly described and informed consent was implied by completing the questionnaire. It took approximately 15 to 20 minutes to complete the questionnaire.

### Data analysis

Statistical analyses were performed using the IBM SPSS Statistics Version 26.0 (IBM Corp., Armonk, NY). Descriptive statistics including means and standard deviations (continuous variables), and frequencies and proportions (categorical variables) were used to report study results where appropriate. Means and standard deviations were compared between sample groups using the independent sample t-test. Proportion comparisons between sample groups were performed using the Pearson’s chi-square test and Fisher’s test. The practice of APNs before and during the COVID-19 was compared using Wilcoxon signed-rank test or McNemar test. Statistical significance was set at *p* < .05 [31].

### Ethical approval

The study protocol was approved by Ethics Committee (SBRE-20-066) and endorsed by ICN Nurse Practitioner/Advanced Practice Nurse Network (NP/APNN). Participants provided informed consent by checking the box of agreeing to participation before proceeding to the survey. Participants completed the survey anonymously and were assured that all responses were kept confidential.

## Results

### Sociodemographic characteristics

A total of 336 participants completed the survey questionnaire; 234 participants were from mainland China and 102 from Hong Kong SAR with 69.6% and 30.4%, respectively. The mean age of participants was 38.32 (*SD* = 7.7), with 14.6% of them were male participants (Table 1). The mean years of experience as a nurse (excluding APN) and an APN were 14.33 (*SD* = 7.19) and 6.55 (*SD* = 5.45) years, respectively. More than half of the participants (64%) had at least a bachelor’s degree. Half of the APNs (50.6%) had a current role as s clinical nurse specialist or nurse practitioners in clinical practice. Majority of the APNs (95.5%) were practicing at the hospital setting and only 1.8% of the APNs were working in outpatient clinics. In this study, 22.6% of the APNs identified cardiovascular disease and/or stroke as their primary practice area.

**Table 1:** Sociodemographic characteristics of study sample (N = 336).

Characteristics	All (n = 336)	Mainland China (n = 234)	Hong Kong SAR (n = 102)	
	Mean (SD)/*N* (%)	Mean (SD)/*n* (%)	Mean (SD)/*n* (%)	*p*
Age*	38.32 (7.7)	35.78 (5.91)	44.14 (8.17)	< .001^a^
Year of experience as a nurse (excluding APN)*	14.33 (7.19)	13.21 (6.7)	16.89 (7.64)	< .001^a^
Year of experience as an APN*	6.55 (5.45)	6.32 (5.02)	7.07 (6.13)	.274^a^
Female	287 (85.4)	212 (90.6)	75 (73.5)	< .001^c^
Highest education level in nursing				< .001^c^
Associate degree	19 (5.7)	19 (8.1)	0 (0.0)	
Bachelor’s degree	215 (64.0)	190 (81.2)	25 (24.5)	
Master’s degree	100 (29.8)	25 (10.7)	75 (73.5)	
Clinical doctoral degree	1 (0.3)	0 (0.0)	1 (1.0)	
Professional diploma	1 (0.3)	0 (0.0)	1 (1.0)	
Current role in clinical practice				< .001^c^
Clinical nurse specialist	170 (50.6)	122 (52.1)	48 (47.0)	
Nurse practitioner/APN	131 (39.0)	104 (44.4)	27 (26.5)	
Certified nurse-midwife	12 (3.6)	0 (0.0)	12 (11.8)	
Nurse consultant	3 (0.9)	1 (0.4)	2 (2.0)	
Other	20 (5.9)	7 (3.0)	13 (12.7)	
Primary practice institution/setting				< .001^c^
Hospital	321 (95.5)	233 (99.6)	88 (86.3)	
Outpatient clinic	6 (1.8)	1 (0.4)	5 (4.9)	
Other	9 (2.7)	0 (0.0)	9 (8.8)	
Primary practice area/direction				< .001^b^
Cardiovascular disease and/or stroke	76 (22.6)	55 (23.5)	21 (20.6)	
Cancer and palliative care	23 (6.8)	21 (9.0)	2 (2.0)	
Geriatric	35 (10.4)	28 (12.0)	7 (6.9)	
Midwifery	20 (6.0)	3 (1.3)	17 (16.7)	
Pediatric	21 (6.3)	16 (6.8)	5 (4.9)	
Nursing education	19 (5.7)	10 (4.3)	9 (8.8)	
Nursing management	32 (9.5)	24 (10.2)	8 (7.8)	
Other	110 (32.7)	77 (32.9)	33 (32.3)	

Note

* Mean (SD), other data are presented as frequency (%).

^a^Independent sample t-test

^b^Pearson’s chi-square test

^c^Fisher test

APN: Advanced Practice Nurse; SAR: Special Administrative Region

### The impacts of the COVID-19 pandemic on the APN practice

As displayed in Table 2, there was a significant difference in providing direct clinical care before and during the COVID-19 pandemic in mainland China, where fewer APNs were involved in direct clinical care during the pandemic (82.9% versus 63.2%) but APNs in Hong Kong SAR reported to have no significant difference in this area of practice (78.4% versus 77.5%). Despite there was a slight decrease in the hours spent on clinical practice during the pandemic when compared to before, APNs in both mainland China [37.01 (*SD* = 12.43) versus 36.69 (*SD* = 12.69)] and Hong Kong SAR [39.96 (*SD* = 10.53) versus 38.96 (*SD* = 11.66)] did not show significant difference in hours spent on clinical practice before and during the pandemic, respectively. Similarly, APNs in mainland China and Hong Kong SAR also reported to have comparable nurse to patient ratio in their practice settings and the number of patients under their care before and during the COVID-19 pandemic (all *ps*>0.05).

**Table 2:** Direct and indirect clinical care provided before and during COVID-19 pandemic.

Characteristics	All (*n* = 336)		Mainland China (*n* = 234)		Hong Kong SAR (*n* = 102)	
	Mean (*SD*)*/N* (%)	*p*	Mean (*SD*)/*n* (%)	*p*	Mean (*SD*)/*n* (%)	*p*
**Direct clinical care**						
Providing direct clinical care		< .001^a^		< .001^a^		1.000^a^
Before COVID-19	274 (81.5)		194 (82.9)		80 (78.4)	
During COVID-19	227 (67.6)		148 (63.2)		79 (77.5)	
Hours spend on clinical practice*						
Before COVID-19	38.05 (11.85)	1.000^b^	37.01 (12.43)	1.000^b^	39.96 (10.53)	1.000^b^
During COVID-19	37.48 (12.35)		36.69 (12.69)		38.96 (11.66)	
Nurse-patient ratio in practice setting*		.070^b^		0.156^b^		0.204^b^
Before COVID-19	0.90 (2.09)		1.03 (2.08)		0.63 (2.09)	
During COVID-19	1.05 (2.28)		1.24 (2.33)		0.69 (2.13)	
Number of patients needed to take care of*		1.000^b^		1.000^b^		1.000^b^
Before COVID-19	12.73 (13.16)		9.39 (6.83)		19.88 (19.30)	
During COVID-19	12.44 (12.85)		9.18 (6.73)		19.51 (18.86)	
**Indirect clinical care**						
Providing indirect clinical care		< .001^a^		.001^a^		.007^a^
Before COVID-19	156 (46.4)		90 (38.5)		66 (64.7)	
During COVID-19	115 (34.2)		62 (26.5)		53 (52.0)	
Hours spent on the following APN role dimensions (indirect clinical care)*						
Educator*		.279^b^		.552^b^		.379^b^
Before COVID-19	11.92 (11.77)		12.53 (12.03)		11.18 (11.54)	
During COVID-19	12.44 (11.30)		9.54 (7.54)		14.55 (13.08)	
Researcher*		.680^b^		1.000^b^		.157^b^
Before COVID-19	5.38 (10.18)		8.55 (12.98)		1.67 (2.33)	
During COVID-19	1.67 (2.37)		1.63 (1.92)		1.69 (2.63)	
Leadership*		.400^b^		.673^b^		.236^b^
Before COVID-19	21.31 (16.24)		21.97 (15.55)		20.69 (17.01)	
During COVID-19	21.88 (16.18)		21.58 (14.71)		22.04 (17.10)	
Consultant*		.339^b^		1.000^b^		.276^b^
Before COVID-19	7.01 (9.79)		7.13 (12.27)		6.92 (7.44)	
During COVID-19	3.34 (3.97)		3.40 (3.41)		3.29 (4.55)	
Other*		.317^b^		1.000^b^		.317^b^
Before COVID-19	12.13 (16.23)		9.17 (13.57)		14.11 (18.31)	
During COVID-19	8.0 (12.06)		1.80 (1.79)		11.44 (14.05)	

Note

*Mean (SD), other data are presented as frequency (%).

^a^ McNemar test

^b^Wilcoxon signed-rank test.

SAR: Special Administrative Region; *SD*: Standard Deviation.

The percentage of APNs providing indirect clinical care decreased significantly during the COVID-19 (34.2%) compared with before the pandemic (46.4%), *p* < .001. Although the time spent as a researcher, consultant, and others reduced during the pandemic, the time spent as an educator and leader increased. Despite these changes, no significant differences were observed regarding the hours spent on each APN role between before and during the COVID-19 in the total study sample, mainland China and Hong Kong SAR.

The majority of participants (79.8%) in this study reported an increased practice-related workload during the pandemic (Table 3). A higher proportion of APNs in mainland China (82.1%) reported an increase in practice-related workload during the COVID-19 pandemic compared to APNs in Hong Kong SAR (74.5%), *p* = .016. The most frequently reported impacts of increased workload among APNs were "increased professional responsibility," followed by "increased psychological distress," and “may affect the quality of patient care”.

**Table 3:** Impacts of the pandemic on practice related workload.

Item	All (N = 336)	Mainland China (*n* = 234)	Hong Kong SAR (*n* = 102)	
	*n* (%)	*n* (%)	*n* (%)	*p*
Has the pandemic increased/decreased practice workload				.016^a^
Stay the same	38 (11.3)	26 (11.1)	12 (11.8)	
Decreased	30 (8.9)	16 (6.8)	14 (13.7)	
Increased	268 (79.8)	192 (82.1)	76 (74.5)	
What are the impacts of increased workload for you?				
Increased professional responsibilities	223 (66.4)	160 (68.4)	63 (61.8)	.238^a^
Increased financial support (payment increase for risk taking)	59 (17.6)	43 (18.4)	16 (15.7)	.551^a^
May affect the quality of patient care	122 (36.3)	71 (30.3)	51 (50.0)	< .001^a^
Increased psychological distress, e.g. anxiety, depression, and/or fear	160 (47.6)	100 (42.7)	60 (58.8)	.007^a^
Increased occupational burnout	102 (30.4)	55 (23.5)	47 (46.1)	< .001^a^
Increased risk for potential errors in practice	109 (32.4)	69 (29.5)	40 (39.2)	.080^a^
Any innovations and/or interventions related to facilitate advanced practice nurse practice since the COVID-19 pandemic began?				.010^a^
No	174 (51.8)	132 (56.4)	42 (41.2)	
Yes	162 (48.2)	102 (43.6)	60 (58.8)	

Note

^a^ Chi-square test; Special Administrative Region.

Nearly half of the participants reported that several innovative measures were implemented to support APN practice since the pandemic began, with higher proportion of participants from Hong Kong SAR (58.8%) compared to those in mainland China (43.6%). APNs from mainland China and Hong Kong SAR responded that there was an increase in the use of telehealth to reduce direct contacts, such as using telehealth for patient assessment and consultation, communication with patient and families through the use of communication software and telephone, and the adoption of online training and education.

### The impacts of the COVID-19 pandemic on patient-focused outcomes

There were changes in patient-focused outcomes during the pandemic. Patient/family satisfaction (14.0%) that was one of the top three patient-focused outcomes before the pandemic, was replaced by psychological well-being (17.3%) during the pandemic. Interestingly, the only focus of care outcome with increased proportions during the COVID-19 pandemic was the psychological well-being of patients when compared with prior to the pandemic, regardless whether in mainland China (9.0% versus 19.2%) or Hong Kong SAR (3.9% versus 12.7%).

### Education training during the COVID-19 pandemic

Nearly all APNs (*n* = 230, 98.3%) from mainland China received education about COVID-19 since the outbreak (Table 4). Meanwhile, the proportion was 72.5% (*n* = 74) in Hong Kong SAR. Among 32 participants from mainland China and Hong Kong who answered that they did not receive education, the top reasons were "busy with other nursing practice" (*n* = 9, 28.1%) and “limited resources for training” (*n* = 11, 34.4%). Individually, three participants from mainland China reported “busy with other nursing practice” while 10 participants from Hong Kong SAR reported “limited resources for training” as the reason for not receiving education about COVID-19 since the outbreak.

**Table 4:** Focus of care outcomes on patients and education training during the COVID-19 pandemic.

Items	All (*N* = 336)	Mainland China (*n* = 234)	Hong Kong SAR (*n* = 102)
	*n* (%)	*n* (%)	*n* (%)
Disease prevention			0.05
Before COVID-19	109 (32.4)	93 (39.7)	16 (15.7)
During COVID-19	124 (36.9)	93 (39.7)	31 (30.4)
Symptom management			0.610
Before COVID-19	143 (42.6)	82 (35.0)	61 (59.8)
During COVID-19	104 (30.9)	63 (26.9)	41 (40.2)
Psychosocial well being			0.507
Before COVID-19	25 (7.4)	21 (9.0)	4 (3.9)
During COVID-19	58 (17.3)	45 (19.2)	13 (12.7)
Patient/family satisfactory			0.931
Before COVID-19	47 (14.0)	37 (15.8)	10 (9.8)
During COVID-19	39 (11.6)	31 (13.2)	8 (7.8)
Cost of care			
Before COVID-19	5 (1.5)	0 (0.0)	5 (4.9)
During COVID-19	2 (0.6)	1 (0.4)	1 (1.0)
Life saving			
Before COVID-19	1 (0.3)	0 (0.0)	1 (1.0)
During COVID-19	1 (0.3)	0 (0.0)	1 (1.0)
Others			0.825
Before COVID-19	6 (1.8)	1 (0.4)	5 (4.9)
During COVID-19	8 (2.4)	1 (0.4)	7 (6.9)
Received education about the COVID-19 since the outbreak			<0.001
No	32 (9.5)	4 (1.7)	28 (27.5)
Yes	304 (90.5)	230 (98.3)	74 (72.5)
Reasons for not receiving education			
Busy with other nursing practice	9 (28.1)	3 (75.0)	6 (21.4)
Limited resources for training	11 (34.4)	1 (25.0)	10 (35.7)
Others	12 (37.5)	0 (0)	12 (42.9)

*Note*. SAR: Special Administrative Region.

Chi-square test

### The challenges, support, and concerns during the COVID-19 pandemic

The greatest personal concern reported by APNs (45.8%) during the COVID-19 pandemic was worries about the “health of family” (Table 5). The biggest concern for clients or healthcare service users related to the COVID-19 pandemic was “health of self “(44.6%), followed by “isolation” (28.6%) and “health of family” (19.3%). The most pressing challenge in advanced nursing practice during the COVID-19 pandemic was the heavy workload (40.2%) and the lack of the latest knowledge about infection prevention and control (30.8%) for APNs in Hong Kong SAR and mainland China, respectively. More than half of the participants (52.7%) reported that psychological support from colleges and/or leaders would be the most supportive factor in facilitating the development of APNs in the current situation.

**Table 5:** The challenges, support, and concerns during COVID-19 pandemic.

Items	All (*N* = 336)	Mainland China (*n* = 234)	Hong Kong SAR (*n* = 102)
	*n (*%)	*n (*%)	*n (*%)
What is your greatest personal concern related to your role in COVID-19?			
Health of self	74 (22.0)	49 (20.9)	25 (24.5)
Health of family	154 (45.8)	109 (46.6)	45 (44.1)
Self-care	13 (3.9)	9 (3.8)	4 (3.9)
Isolation	57 (17.0)	48 (20.5)	9 (8.8)
Job burnout	27 (8.0)	13 (5.6)	14 (13.7)
Social prejudice	6 (1.8)	5 (2.1)	1 (1.0)
Government policy	1 (0.3)	0 (0.0)	1 (1.0)
Others	4 (1.2)	1 (0.4)	3 (2.9)
What is the biggest concern that your clients / service users have identified related to COVID-19?			
Health of self	150 (44.6)	107 (45.7)	43 (42.1)
Health of family	65 (19.3)	46 (19.7)	19 (18.6)
Isolation	96 (28.6)	65 (27.8)	31 (30.4)
Social prejudice	22 (6.5)	16 (6.8)	6 (5.9)
Government policy	1 (0.3)	0 (0.0)	1 (1.0)
Others	2 (0.6)	0 (0.0)	2 (2.0)
What is the most pressing challenge in your advanced nursing practice in the COVID-19 pandemic situation?			
Lack of latest knowledge about COVID-19 pandemic	43 (12.8)	26 (11.1)	17 (16.7)
Lack of latest knowledge about infection prevention and control	88 (26.2)	72 (30.8)	16 (15.7)
Lack of sufficient high-quality and appropriate personal protective equipment	52 (15.5)	37 (15.8)	15 (14.7)
Heavy workload	109 (32.4)	68 (29.1)	41 (40.2)
Reimbursement issues	7 (2.1)	7 (3.0)	0 (0.0)
Lack of support from family and/or friends	7 (2.1)	5 (2.1)	2 (2.0)
Lack of support from public	23 (6.8)	19 (8.1)	4 (3.9)
Others	7 (2.1)	0 (0.0)	7 (6.9)
What support do you think would most facilitate the development of APN in the current situation?			
Psychological support from family and/or friends	109 (32.4)	91 (38.9)	18 (17.6)
Psychological support from colleagues and/or leader	177 (52.7)	127 (54.3)	50 (49.0)
Mental health counselling	63 (18.8)	50 (21.4)	13 (12.7)
Effective cooperation with other healthcare professionals	145 (43.2)	101 (43.2)	44 (43.1)
Additional finical support	128 (38.1)	106 (45.3)	22 (21.6)
Public’s adherence to the latest public health advices	88 (26.2)	66 (28.2)	22 (21.6)
Health policies and public health measures	116 (34.5)	76 (32.5)	40 (39.2)
Policies for expanding the nursing workforce	124 (36.9)	92 (39.3)	32 (31.4)
Others	1 (0.3)	0 (0.0)	1 (1.0)

*Note*. SAR: Special Administrative Region.

## Discussion

This study investigated the impacts of COVID-19 on the practice of APNs, patient-focused outcomes, and explored their needs, concerns, and challenges during the pandemic. To respond to the COVID-19 pandemic, the role of APNs in clinical practice has been largely changed to address the needs of patients with COVID-19, their family members, and healthcare providers. APNs play an important role in providing individualized care and improving the quality of care in the current healthcare system. Support from colleagues plays an important role for APNs in managing the impact of the COVID-19 pandemic. Investigating APNs’ experiences and roles during the pandemic can enhance our understanding of responsibilities and concerns of APNs and inform future service provision and support.

In line with several recent studies [10, 32, 33], this study demonstrates that the COVID-19 pandemic had resulted in changes in the practice of APNs. A significant decrease in the number of participants providing direct and indirect clinical care was observed in our study. Despite the number of patients needed to be taken care of being reduced during the pandemic, the practice-related workload had increased among the APNs. In our study, 66.4% of participants perceived that their professional responsibilities had increased with the increased practice-related workload. This finding is consistent with an exploratory qualitative study which was conducted in Canada, where nurse practitioners reported to have increased workload as they embraced many roles and mitigated the spread of the COVID-19 at the same time [34]. In another qualitative study, healthcare professionals perceived they had increased professional responsibilities as they were most needed by their country and people, and saving lives were their duty at this particular time [35]. Meanwhile in the UK, 43% of the APNs in a cross-sectional mixed-methods survey reported working more overtime during the pandemic [11]. The increased practice-related workload might have caused some adverse psychological effects, with nearly half of the participants reporting increased psychological distress in this study. A recent cross-sectional study from 33 countries reported that 64.5% of participants consisted of frontline health providers were considered at risk of psychological distress during the COVID-19 pandemic [36]. A study conducted in mainland China by Leng et al. [37] reported 22.2% of nurses had some degree of perceived stress. Being a frontline worker was associated with higher level of fear during the COVID-19 pandemic [38]. APNs who face increased workload and psychological distress are prone to burnout. A recent prospective longitudinal survey by Maunder et al. [39], reported that during the COVID-19 pandemic in year 2021, nurses experienced the highest levels of psychological distress compared to other healthcare professionals; and the rate of high emotional exhaustion (burnout) was highest among nurses. A survey in the US found that nearly 90% of APNs had a moderate or high burnout during the COVID-19 pandemic [40]. Maunder et al. [39] highlighted that the levels of emotional exhaustion continued to be higher than those measured levels in the hospitals pre-pandemic even though emotional exhaustion decreased as the case rate of COVID-19 declined. The APNs in our study perceived that the increased practice-related workload affected the quality of patient care. In addition, burnout has also been found to be significantly associated with poor patient safety outcomes (e.g., medical errors) [41].

Both workload perception and burnout were found to have moderate to strong intercorrelations with an increased intention to leave the job among nurses [42]. Evidence has shown that a better work environment reduces burnout and intention to leave, and the favorable work environment includes strong nurse managers, leadership, shared responsibility in nurse-patient relations (collegiality), supportive environment in teaching and learning, adequate staffing and resources [43]. Therefore, managers and policymakers are urged to take actions in improving the work environments for APNs not only during a public health emergency, but ongoing efforts are required to reduce burnout and intention to leave among APNs, and provide quality of care to patients. Also, findings from our study suggest that hospitals or healthcare organizations should offer health monitoring and mental support to APNs in order to address their work-related concerns and share coping strategies during an unprecedented period of public health threat in the future.

Although we did not look into redeployment in this study, the phenomenon of “being pulled” or “floating” to other units is not new in nursing practice. In fact, it was amplified during the COVID-19 pandemic [44] to address staffing shortages. Nurses’ psychological health was significantly affected by the unfamiliarity of the work environment and taking care of patients outside of their regular acuity levels [37]. Kennedy et al. [44] suggests that staff sharing across similar units with rotation, providing adequate orientation, and equipping nurses with training to provide safe patient care are some strategies to support staff wellbeing as well as quality patient care. These strategies can support the preparedness of APNs and all frontline nurses in any circumstances. The focus of care outcomes for patients also changed as more APNs focused on disease prevention and psychosocial well-being during the pandemic compared to before the pandemic. The change of their practice might be resulted from scheduling changes, increased workload, exhaustion due to protective gear, and lack of staff [35]. Furthermore, APNs were on the frontline of treatment during the COVID-19 pandemic, which required them to remain current with treatments and updates to patient education to promote prevention of disease and the spread of infection [12].

Despite the increased practice-related workload and psychological distress caused, there are some positive findings. Over 90% of participants had received education about the COVID-19. Nearly all participants in mainland China had received education about COVID-19. These findings suggest that early preparation should be made to provide relevant education to healthcare professionals in the face of future infectious disease pandemics. Over half of the participants reported that some innovative measures related to facilitating APN practice were implemented since the COVID-19 pandemic began. Innovative measures related to facilitating APN practice and education in clinical practice can help prepare APNs better understand the prevention and treatment of the pandemic and provide direct or indirect patient care. In this study, one of the innovative measures included telehealth, aligning with the global call for action by nurse researchers to offer technological decision-making in clinical practice and integrate of technology and informatics into current and future care delivery [45, 46]. In addition, the use of technology such as videoconferencing allows real-time communication without being in the same location or face-to-face encounters, closing the gaps related to accessibility, transportation, and other limitations. However, Schroeder [47] mentioned that there are some concerns related to the virtual world of technology such as inability to establish good rapport with the patients, failure to detect nonverbal cues, privacy concerns, and technical issues. In these situations, APNs must be prepared, educated, and knowledgeable to incorporate technology into their scope of practice, but at the same time provide safe care and best practices. The expanded use of telehealth and virtual care also indicates the need for new rules and regulations in this field.

Most of the participants perceived their health and their families’ health as the greatest concern during the pandemic. A recent study in the UK also found that APNs were concerned about protecting their own health, and that of their family members. Their safety concerns may cause a higher intention to leave their jobs during the pandemic [11]. Also, over half of the participants stated a need for psychological support from their colleagues and/or leaders. A previous cross-sectional study on nurses in Hubei, mainland China reported that several factors associated with the willingness to volunteer during the COVID-19 pandemic including adequate training and psychological support [48]. These challenges implied the urgent need for applying more innovations related to facilitating APN practice, and continuing education and training about the pandemic for APNs [49]. In addition, the findings highlight the needs for future interventions to provide psychological assistance to APNs and enable them to perform patient care effectively.

Our study has several limitations. First, a cross-sectional design with snowball sampling may cause selection bias and the participants in our study were only from mainland China and Hong Kong SAR, limiting the generalizability of the study to a broader population. Nearly 96% of participants were working in hospitals, which could limit our understanding of the practice of APNs working in other settings. In terms of questionnaire design, a more explicit time range should be provided for the items about the practice issues before and during the COVID-19 pandemic (e.g., one month before the COVID-19 pandemic) to reduce the potential recall bias among participants. Nevertheless, this study focused on APNs, who play a key role in addressing global health challenges and face unique changes during the COVID-19 pandemic.

### Implications to education, research, practice, and health policy

Our findings highlight the importance of mandatory education about COVID-19 for APNs, even though over 90% of participants in our study reported receiving such education during the pandemic. Continuing education and training are essential for APNs to be able to provide highest quality of care, protect vulnerable populations, and able to develop innovative solutions during ongoing public health crisis [12]. Therefore, organizations should make it a priority to keep all APNs up-to-date on evidence-based practice to prepare them in facing future public health crises. The large number of COVID-19 patients put a significant strain on healthcare resources, limiting educational and training opportunities. Hence, new methods are required to ensure the continuity of learning and skill acquisition [50]. A greater need for virtual conference and training opportunities is necessary among healthcare providers during the COVID-19 pandemic. Besides the feasibility of incorporating latest information and developments, virtual training programs allow APNs to participate and engage in learning activities without living their practice sites [51]. Future studies may be needed to assess the effectiveness of virtual education and training platforms compared to traditional face-to-face formats, which could help prepare for the continuation of essential training and educational activities among APNs, even during public health crises like pandemics.

The APN programs in mainland China are relatively new as above-mentioned, it is necessary to explore the acceptance among learners in adopting new ways of learning using the technology and it affects APNs towards learning. This will be helpful in providing insights to academic leaders to develop nursing curricula that can be delivered through different methods regardless if face-to-face or virtual. Besides, research on psychological distress faced by APNs should be conducted to explore its factors and evaluate the long-term effects. Effective strategies can be implemented to mitigate psychological distress in APNs based on the findings.

The COVID-19 pandemic has placed significant physical and mental health burdens on APNs. Our study findings revealed increased practice-related workload during the unprecedented time, and one of its impacts was increased psychological distress. If this situation is left unaddressed, it could contribute to burnout among APNs. In long-term, this could affect the work sustainability and retention of APNs. A survey by Kelly et al. [52] revealed that for every single-unit increase in burnout experienced by nurses, there was a corresponding 12 percent increase in the likelihood of those nurses leaving the healthcare system. Hence, organizations need to engage in proactive measures to improve work sustainability and retention among APNs. Policy makers and employers need to engage in proactive measures such as evaluating and optimizing the work environments of APNs to mitigate negative health outcomes during the COVID-19 pandemic [53], in turn, enhance the long-term sustainability and retention of the APN workforce. To prepare APNs in redeployment to other units in the ever-changing healthcare needs and in facing the challenges of other public health threats in the future, APNs must have versatility and ability to adapt. Leaders should consider scheduled rotation of APNs to other units, with minimum hours per month to refresh their skills and knowledge in different areas of healthcare.

## Conclusions

The findings indicate that the roles and responsibilities of APNs in mainland China and Hong Kong SAR have shown similarities, and these roles have undergone significant changes in response to the COVID-19 pandemic. The changes suggest that APNs have faced an increased practice-related workload and psychological distress during this period. To enhance their professional practices, it is crucial to provide education on COVID-19, implement innovative measures to support APN practice, and understand APNs’ concerns and challenges in providing patient care. Our findings help to inform interventions to better prepare APNs for the burden and stress caused by the pandemic and create supportive policies to ensure a safe and efficient work environment for them. Healthcare organizations are recommended to offer long-term health monitoring and mental support to APNs to relieve their psychological distress associated with increased practice-related workload at this time.

## References

[1] Li J, Lai S, Gao GF, Shi W. The emergence, genomic diversity and global spread of SARS-CoV-2. Nature. 2021 Dec;600(7889):408–418. doi: 10.1038/s41586-021-04188-6 34880490

[2] Muralidar S, Ambi SV, Sekaran S, Krishnan UM. The emergence of COVID-19 as a global pandemic: Understanding the epidemiology, immune response and potential therapeutic targets of SARS-CoV-2. Biochimie. 2020 Dec;179:85–100. doi: 10.1016/j.biochi.2020.09.018 32971147 PMC7505773

[3] World Health Organization. COVID-19 epidemiological update– 22 December 2023. 2023. Available from: https://www.who.int/publications/m/item/covid-19-epidemiological-update—22-december-2023

[4] ICN. The greatest threat to global health is the workforce shortage”—International Council of Nurses International Nurses Day demands action on investment in nursing, protection and safety of nurses. 2022. Available from: https://www.icn.ch/news/greatest-threat-global-health-workforce-shortage-international-council-nurses-international

[5] Ferland L, Carvalho C, Gomes Dias J, Lamb F, Adlhoch C, Suetens C, et al. Risk of hospitalization and death for healthcare workers with COVID-19 in nine European countries, January 2020-January 2021. J Hosp Infect. 2022 Jan;119:170–174. doi: 10.1016/j.jhin.2021.10.015 34752802 PMC8665668

[6] Al Thobaity A, Alshammari F. Nurses on the frontline against the COVID-19 pandemic: an integrative review. Dubai Medical Journal. 2020 Aug 26:1–6. doi: 10.1159/000509361

[7] Galletta M, Piras I, Finco G, Meloni F, D’Aloja E, Contu P, et al. Worries, preparedness, and perceived impact of Covid-19 pandemic on nurses’ mental health. Front Public Health. 2021 May 26;9:566700. doi: 10.3389/fpubh.2021.566700 34123979 PMC8187773

[8] Zipf AL, Polifroni EC, Beck CT. The experience of the nurse during the COVID-19 pandemic: A global meta-synthesis in the year of the nurse. J Nurs Scholarsh. 2022 Jan;54(1):92–103. doi: 10.1111/jnu.12706 34738314 PMC8662101

[9] Stucky CH, Brown WJ, Stucky MG. COVID 19: An unprecedented opportunity for nurse practitioners to reform healthcare and advocate for permanent full practice authority. Nurs Forum. 2021 Jan;56(1):222–227. doi: 10.1111/nuf.12515 33047352 PMC7675696

[10] Kleinpell R, Myers CR, Schorn MN, Likes W. Impact of COVID-19 pandemic on APRN practice: Results from a national survey. Nurs Outlook. 2021 Sep-Oct;69(5):783–792. doi: 10.1016/j.outlook.2021.05.002 34176669 PMC8112385

[11] Wood E, King R, Senek M, Robertson S, Taylor B, Tod A, et al. UK advanced practice nurses’ experiences of the COVID-19 pandemic: a mixed-methods cross-sectional study. BMJ Open. 2021 Mar 16;11(3):e044139. doi: 10.1136/bmjopen-2020-044139 33727270 PMC7969757

[12] Diez-Sampedro A, Gonzalez A, Delgado V, Flowers M, Maltseva T, Olenick M. COVID-19 and advanced practice registered nurses: frontline update. J Nurse Pract. 2020 Sep;16(8):551–555. doi: 10.1016/j.nurpra.2020.06.014 32837398 PMC7301091

[13] Holroyd E, McNaught C. The SARS crisis: reflections of Hong Kong nurses. Int Nurs Rev. 2008 Mar;55(1):27–33. doi: 10.1111/j.1466-7657.2007.00586.x 18275532

[14] Shih FJ, Turale S, Lin YS, Gau ML, Kao CC, Yang CY, et al. Surviving a life-threatening crisis: Taiwan’s nurse leaders’ reflections and difficulties fighting the SARS epidemic. J Clin Nurs. 2009 Dec;18(24):3391–400. doi: 10.1111/j.1365-2702.2008.02521.x 19207797

[15] Hung LS. The SARS epidemic in Hong Kong: what lessons have we learned? J R Soc Med. 2003 Aug;96(8):374–8. doi: 10.1177/014107680309600803 12893851 PMC539564

[16] Cooper BS, Fang LQ, Zhou JP, Feng D, Lv H, Wei MT, et al. Transmission of SARS in three Chinese hospitals. Trop Med Int Health. 2009 Nov;14 Suppl 1(Suppl 1):71–8. doi: 10.1111/j.1365-3156.2009.02346.x 19814763 PMC7169696

[17] Liu H, Liehr P. Instructive messages from Chinese nurses’ stories of caring for SARS patients. J Clin Nurs. 2009 Oct;18(20):2880–7. doi: 10.1111/j.1365-2702.2009.02857.x 19747256

[18] Chen R, Chou KR, Huang YJ, Wang TS, Liu SY, Ho LY. Effects of a SARS prevention programme in Taiwan on nursing staff’s anxiety, depression and sleep quality: a longitudinal survey. Int J Nurs Stud. 2006 Feb;43(2):215–25. doi: 10.1016/j.ijnurstu.2005.03.006 15927185 PMC7094227

[19] Wong FK, Wong AK. Advanced practice nursing in Hong Kong and mainland China. Advanced practice nursing leadership: A global perspective. 2020:105–14.

[20] Wong FK, Peng G, Kan EC, Li Y, Lau AT, Zhang L, et al. Description and evaluation of an initiative to develop advanced practice nurses in mainland China. Nurse Educ Today. 2010 May;30(4):344–9. doi: 10.1016/j.nedt.2009.09.004 19819051

[21] Chun CK, Wong FK, Wang SL, Chen W. Examining advanced nursing practice in Hong Kong and Guangzhou. Int J Nurs Sci. 2021 Mar 5;8(2):190–198. doi: 10.1016/j.ijnss.2021.03.001 33997133 PMC8105546

[22] Mao X, Yang Q, Li X, Chen X, Guo C, Wen X, et al. An illumination of the ICN’s core competencies in disaster nursing version 2.0: Advanced nursing response to COVID-19 outbreak in China. J Nurs Manag. 2021 Apr;29(3):412–420. doi: 10.1111/jonm.13195 33107099

[23] Chan DS, Lee DT, Chair SY, Fung SY, Chan EL, Chan CW. A qualitative study on the roles and responsibilities of nurse consultants in Hong Kong. Int J Nurs Pract. 2014 Oct;20(5):475–81. doi: 10.1111/ijn.12181 24118297

[24] Newall F, Twomey B, Lima S. Advanced practice nursing–Promoting organisation clarity and connectedness: a mixed methods approach. Collegian. 2018 Feb 1;25(1):97–103. doi: 10.1016/j.colegn.2017.04.003

[25] ICN. Guidelines on advanced practice nursing. 2020. Available from: https://icn.ch/system/files/documents/2020-04/ICN_APN%20Report_EN_WEB.pdf

[26] Almanasreh E, Moles R, Chen TF. Evaluation of methods used for estimating content validity. Res Social Adm Pharm. 2019 Feb;15(2):214–221. doi: 10.1016/j.sapharm.2018.03.066 29606610

[27] Lynn MR. Determination and quantification of content validity. Nurs Res. 1986 Nov-Dec;35(6):382–5. 3640358

[28] Polit DF, Beck CT. The content validity index: are you sure you know what’s being reported? Critique and recommendations. Res Nurs Health. 2006 Oct;29(5):489–97. doi: 10.1002/nur.20147 16977646

[29] Rosenthal R. Parametric measures of effect size. In Cooper H, Hedges LV, editors. The handbook of research synthesis. Russell Sage Foundation; 1994. pp. 231–244.

[30] Faul F, Erdfelder E, Lang AG, Buchner A. G*Power 3: a flexible statistical power analysis program for the social, behavioral, and biomedical sciences. Behav Res Methods. 2007 May;39(2):175–91. doi: 10.3758/bf03193146 17695343

[31] Fisher RA. Statistical methods for research workers. Breakthroughs in statistics: Methodology and Distribution. New York, NY: Springer New York; 1970. pp. 66–70.

[32] Eftekhar Ardebili M, Naserbakht M, Bernstein C, Alazmani-Noodeh F, Hakimi H, Ranjbar H. Healthcare providers experience of working during the COVID-19 pandemic: A qualitative study. Am J Infect Control. 2021 May;49(5):547–554. doi: 10.1016/j.ajic.2020.10.001 33031864 PMC7536124

[33] White JH. A phenomenological study of nurse managers’ and assistant nurse managers’ experiences during the COVID-19 pandemic in the United States. J Nurs Manag. 2021 Sep;29(6):1525–1534. doi: 10.1111/jonm.13304 33690928 PMC8237030

[34] McGilton KS, Krassikova A, Boscart V, Sidani S, Iaboni A, Vellani S, et al. Nurse practitioners rising to the challenge during the Coronavirus Disease 2019 pandemic in long-term care homes. Gerontologist. 2021 Jun 2;61(4):615–623. doi: 10.1093/geront/gnab030 33659982 PMC7989234

[35] Liu Q, Luo D, Haase JE, Guo Q, Wang XQ, Liu S, et al. The experiences of health-care providers during the COVID-19 crisis in China: a qualitative study. Lancet Glob Health. 2020 Jun;8(6):e790–e798. doi: 10.1016/S2214-109X(20)30204-7 32573443 PMC7190296

[36] Collins C, Clays E, Van Poel E, Cholewa J, Tripkovic K, Nessler K, et al. Distress and Wellbeing among General Practitioners in 33 Countries during COVID-19: Results from the Cross-Sectional PRICOV-19 Study to Inform Health System Interventions. Int J Environ Res Public Health. 2022 May 6;19(9):5675. doi: 10.3390/ijerph19095675 35565070 PMC9101443

[37] Leng M, Wei L, Shi X, Cao G, Wei Y, Xu H, et al. Mental distress and influencing factors in nurses caring for patients with COVID-19. Nurs Crit Care. 2021 Mar;26(2):94–101. doi: 10.1111/nicc.12528 33448567

[38] Chair SY, Chien WT, Liu T, Lam L, Cross W, Banik B, et al. Psychological distress, fear and coping strategies among Hong Kong people during the COVID-19 Pandemic. Curr Psychol. 2023;42(3):2538–2557. doi: 10.1007/s12144-021-02338-7 34690470 PMC8527280

[39] Maunder RG, Heeney ND, Hunter JJ, Strudwick G, Jeffs LP, Ginty L, et al. Trends in burnout and psychological distress in hospital staff over 12 months of the COVID-19 pandemic: a prospective longitudinal survey. J Occup Med Toxicol. 2022 May 25;17(1):11. doi: 10.1186/s12995-022-00352-4 35614505 PMC9132565

[40] Stallter C, Gustin TS. Evaluating advanced practice nurses’ burnout and potential helping modalities. J Nurse Pract. 2021 Nov-Dec;17(10):1297–1299. doi: 10.1016/j.nurpra.2021.07.003 35095350 PMC8782823

[41] Hall LH, Johnson J, Watt I, Tsipa A, O’Connor DB. Healthcare Staff Wellbeing, Burnout, and Patient Safety: A Systematic Review. PLoS One. 2016 Jul 8;11(7):e0159015. doi: 10.1371/journal.pone.0159015 27391946 PMC4938539

[42] Phillips C. Relationships between workload perception, burnout, and intent to leave among medical-surgical nurses. Int J Evid Based Healthc. 2020 Jun;18(2):265–273. doi: 10.1097/XEB.0000000000000220 32141948

[43] Nantsupawat A, Kunaviktikul W, Nantsupawat R, Wichaikhum OA, Thienthong H, Poghosyan L. Effects of nurse work environment on job dissatisfaction, burnout, intention to leave. Int Nurs Rev. 2017 Mar;64(1):91–98. doi: 10.1111/inr.12342 27882573

[44] Kennedy E, Kennedy P, Hernandez J, Shakoor K, Munyan K. Understanding Redeployment During the COVID-19 Pandemic: A Qualitative Analysis of Nurse Reported Experiences. SAGE Open Nurs. 2022 Jul 21;8:23779608221114985. doi: 10.1177/23779608221114985 35899038 PMC9310283

[45] Clipper B. The Influence of the COVID-19 Pandemic on Technology: Adoption in Health Care. Nurse Lead. 2020 Oct;18(5):500–503. doi: 10.1016/j.mnl.2020.06.008 32837346 PMC7324321

[46] Dykes S, Chu CH. Now more than ever, nurses need to be involved in technology design: lessons from the COVID-19 pandemic. J Clin Nurs. 2021 Apr;30(7–8):e25–e28. doi: 10.1111/jocn.15581 33289230 PMC7753642

[47] Schroeder RA. Adaptation or Revolution: Telemental Health and Advanced Practice Psychiatric Nursing During COVID-19. J Am Psychiatr Nurses Assoc. 2022 May-Jun;28(3):241–248. doi: 10.1177/1078390320970638 33164642

[48] Gan X, Shi Z, Chair SY, Cao X, Wang Q. Willingness of Chinese nurses to practice in Hubei combating the coronavirus disease 2019 epidemic: A cross-sectional study. J Adv Nurs. 2020 Aug;76(8):2137–2150. doi: 10.1111/jan.14434 32449187 PMC7283855

[49] Rathnayake S, Dasanayake D, Maithreepala SD, Ekanayake R, Basnayake PL. Nurses’ perspectives of taking care of patients with Coronavirus disease 2019: A phenomenological study. PLoS One. 2021 Sep 3;16(9):e0257064. doi: 10.1371/journal.pone.0257064 34478482 PMC8415609

[50] Boutros P, Kassem N, Nieder J, Jaramillo C, von Petersdorff J, Walsh FJ, et al. Education and Training Adaptations for Health Workers during the COVID-19 Pandemic: A Scoping Review of Lessons Learned and Innovations. Healthcare (Basel). 2023 Nov 4;11(21):2902. doi: 10.3390/healthcare11212902 37958046 PMC10649637

[51] Smith TS, Holland AC, White T, Combs B, Watts P, Moss J. A Distance Accessible Education Model: Teaching Skills to Nurse Practitioners. J Nurse Pract. 2021 Sep;17(8):999–1003. doi: 10.1016/j.nurpra.2021.05.018 35165528 PMC8828427

[52] Kelly LA, Gee PM, Butler RJ. Impact of nurse burnout on organizational and position turnover. Nurs Outlook. 2021 Jan-Feb;69(1):96–102. doi: 10.1016/j.outlook.2020.06.008 33023759 PMC7532952

[53] Havaei F, Ma A, Staempfli S, MacPhee M. Nurses’ workplace conditions impacting their mental health during COVID-19: A cross-sectional survey study. Healthcare (Basel). 2021 Jan 16;9(1):84. doi: 10.3390/healthcare9010084 33467080 PMC7830057

